# New Italian Review on Hygiene

**Published:** 1890-01

**Authors:** 


### New Italian Review on Hygiene.

A further indication of the progress which is being made in matters relating to public health and sanitary science in the kingdom of Italy is afforded by the promise of the issue next month of the first number of a Review on Hygiene, which will be compiled under the direction of Dr. Eugenio Fazio. The subjects to be dealt with are as follows: Biology, bacteriology, infection and sanitary police, the natural sciences as applied to public health, demography, statistics and sanitary engineering. We are glad to find that the Review will emanate from Naples, for the southern portion of the Italian peninsula stands, we fear, much in need of an educational movement in the direction of hygiene. For this reason, too, the Review has our good wishes.

